# Mitochondrial Genome Sequences of *Diorhabda carinata* and *Diorhabda carinulata*, Two Beetle Species Introduced to North America for Biological Control

**DOI:** 10.1128/MRA.00690-19

**Published:** 2019-08-29

**Authors:** A. R. Stahlke, A. Z. Ozsoy, D. W. Bean, P. A. Hohenlohe

**Affiliations:** a Institute for Bioinformatics and Evolutionary Studies, University of Idaho, Moscow, Idaho, USA; b Bioinformatics and Computational Biology Graduate Program, University of Idaho, Moscow, Idaho, USA; c Department of Biological Sciences, Colorado Mesa University, Grand Junction, Colorado, USA; d Colorado Department of Agriculture, Palisade, Colorado, USA; Vanderbilt University

## Abstract

We announce the complete circularized mitochondrial genome assemblies of Diorhabda carinata and Diorhabda carinulata, beetle species introduced to North America for the biological control of invasive shrubs of the genus Tamarix L. (Tamaricaceae). The assemblies (16,232 and 16,298 bp, respectively) each comprise 13 protein-coding genes, 22 tRNAs, two rRNAs, and a noncoding region.

## ANNOUNCEMENT

The tamarisk beetle, a cryptic species complex in the genus Diorhabda (Coleoptera: Chrysomelidae), originated from Eurasia and was introduced to North America for the biological control of invasive Tamarix spp. (1). To better understand evolution within this group of beetles, we assembled and annotated the mitochondrial genomes of Diorhabda carinata and Diorhabda carinulata, which are the only introduced *Diorhabda* species that are sympatric in their native ranges (1).

Bean and colleagues (2) examined evolutionary relationships among introduced *Diorhabda* spp. This study revealed polyphyly based on the cytochrome oxidase subunit I mitochondrial gene, while analysis of nuclear loci (amplified fragment length polymorphism analysis) grouped samples into four clades corresponding to their morphospecies designations. Additionally, D. carinata readily hybridizes with Diorhabda sublineata and Diorhabda elongata under laboratory conditions without a reduction in fecundity (2, 3) and appears to do so in the field (4). *D. carinulata* failed to produce stable hybrids with the other three clades (2). These results warrant further work to determine the possible influence of introgression, mitochondrial selection, or sex-biased dispersal patterns (5).

For this study, we used a single male from full-sibling inbred lines developed from continuous cultures of each species at the Palisade Insectary, Palisade, CO. We produced a 26-generation inbred line of *D. carinata* originating from Qarshi, Uzbekistan (38.86°N, 65.72°E). A five-generation inbred line of *D. carinulata* was produced from a laboratory culture established from field-collected beetles in Lovelock, NV (40.02°N, 118.52°W), where *D. carinulata* from Fukang, China (44.17°N, 87.98°E), was released in 2001. For *D. carinata,* we dissected the testes and extracted DNA with a MagAttract high-molecular-weight (HMW) DNA kit (Qiagen). For *D. carinulata*, we dissected the head, thorax, and testes using a DNeasy blood and tissue kit (Qiagen). We constructed whole-genome shotgun sequencing libraries using the NEBNext Ultra II DNA library prep kit for both species. The *D. carinata* library was sequenced on a HiSeq 4000 platform (Illumina) to produce paired 150-bp reads. The *D. carinulata* library was sequenced on a MiSeq platform (Illumina) using v3 reagents to produce paired 300-bp reads.

We trimmed adapters from raw reads using Sickle 1.33 (6). With 44,665,534 reads for *D. carinata* and 28,532,798 reads from *D. carinulata,* we used NOVOPlasty 2.7.2 (7) to assemble each mitochondrial genome. The Diabrotica barberi mitochondrion (GenBank accession number KF669870) was used to seed the *D. carinata* assembly. Then, we used the *D. carinata* assembly to seed the *D. carinulata* assembly. Annotations were performed with MITOS2 (last modified 16 June 2017; Git hash 6b33f95) (8) using RefSeq 63 Metazoa and the invertebrate genetic code.

From the *D. carinata* and *D. carinulata* reads, we assembled one circularized mitochondrion assembly per species of lengths 16,232 and 16,298 bp, average coverages of 3,323× and 3,846×, and G+C contents of 22.1% and 21.1%, respectively. Annotations of both genomes comprise 13 protein-coding genes, 22 tRNAs, two rRNAs, and a noncoding region (d-loop). We did not identify light origin (OL) in either assembly. The assembly sizes, G+C contents, and annotations are consistent with those of other chrysomelid mitochondrial genomes (9–13).

### Data availability.

Raw reads and mitochondrial DNA (mtDNA) genome sequences for *D. carinata* and *D. carinulata* have been deposited in GenBank under accession numbers PRJNA513507, MK359256, and MK359257.
